# Collagen Type II-Based Injectable Materials for In situ Repair and Regeneration of Articular Cartilage Defect

**DOI:** 10.34133/bmr.0072

**Published:** 2024-08-30

**Authors:** Zhen Zhang, Xu Hu, Min Jin, Yulei Mu, Huiqun Zhou, Cheng Ma, Liang Ma, Bangheng Liu, Hang Yao, Ye Huang, Dong-An Wang

**Affiliations:** ^1^ Department of Biomedical Engineering, City University of Hong Kong, Kowloon, Hong Kong SAR.; ^2^ Karolinska Institutet Ming Wai Lau Centre for Reparative Medicine, HKSTP, Sha Tin, Hong Kong SAR.; ^3^ School of Chemistry and Chemical Engineering, Yangzhou University, Yangzhou, China.; ^4^ Knee Preservation Clinical and Research Center, Beijing Jishuitan Hospital, Beijing, China.; ^5^ Center for Neuromusculoskeletal Restorative Medicine, HKSTP, Shatin, Hong Kong SAR.

## Abstract

Repairing and regenerating articular cartilage defects (ACDs) have long been challenging for physicians and scientists. The rise of injectable materials provides a novel strategy for minimally invasive surgery to repair ACDs. In this study, we successfully developed injectable materials based on collagen type II, achieving hyaline cartilage repair and regeneration of ACDs. Analysis was conducted on the regenerated cartilage after materials injection. The histology staining demonstrated complete healing of the ACDs with the attainment of a hyaline cartilage phenotype. The biochemical and biomechanical properties are similar to the adjacent native cartilage without noticeable adverse effects on the subchondral bone. Further transcriptome analysis found that compared with the Native cartilage adjacent to the defect area, the Regenerated cartilage in the defect area repaired with type II collagen-based injection materials showed changes in cartilage-related pathways, as well as down-regulation of T cell receptor signaling pathways and interleukin-17 signaling pathways, which changed the immune microenvironment of the ACD area. Overall, these findings offer a promising injectable approach to treating ACDs, providing a potential solution to the challenges associated with achieving hyaline cartilage in situ repair and regeneration while minimizing damage to the surrounding cartilage.

## Introduction

As one of the major orthopedic diseases, articular cartilage defects (ACDs) can be caused by trauma, aging, and other factors. ACDs lead to pain, impaired joint function, decreased quality of life, and an increased risk of developing osteoarthritis (OA) [1]. Due to the absence of nerves and blood vessels in articular cartilage, its intrinsic ability to self-heal is limited, presenting a important challenge in repairing ACDs. Unfortunately, current cartilage repair techniques often result in fibrocartilage repair, failing to achieve hyaline cartilage repair [2–4]. Moreover, these techniques necessitate the excision of surrounding healthy cartilage during surgery, resulting in increased surgical trauma and prolonged patient recovery time [4,5]. With the development of injectable materials, minimally invasive surgery has become achievable [6]. Minimally invasive surgery has small incisions, which are beneficial to reducing postoperative infection, alleviating postoperative pain, and patients’ postoperative recovery, which highlights the urgency and societal value of developing injectable articular cartilage repair materials. Hydrogels have emerged as a forefront area of research in cartilage repair materials because of their high water content, similar to cartilage tissue [7]. Hydrogels made from synthetic polymers such as poly (vinyl alcohol), polyethylene glycol, and polyacrylamide can exhibit excellent mechanical strength [8–10]. However, addressing the issue of bio-inertness remains a challenge [11]. As a result, natural macromolecules with favorable biocompatibility, including polysaccharides such as chondroitin sulfate [12], hyaluronic acid, chitosan, and alginate, as well as proteins and peptides, have garnered substantial attention [6]. Nevertheless, these materials still possess drawbacks such as poor mechanical properties and rapid degradation. It is important to note that existing research on these materials refers to the regenerated cartilage as “hyaline-like cartilage” rather than actual hyaline cartilage, indicating that achieving authentic hyaline cartilage regeneration with these materials is still a distant goal. Wei et al. have comprehensively summarized the specific findings in a recent review [11]. Therefore, achieving in situ injectability of materials and the correct hyaline cartilage phenotype of regeneration tissue are the 2 main objectives of this study.

The development and application of natural “glues” have gained substantial attention due to their super biocompatibility and biodegradability [6,13]. One such example is fibrin glue (F-gel), which is inspired by the adhesive properties of blood clots and has been approved for use in humans as a biological adhesive and hemostatic agent [14,15]. To achieve injectability of the material, this study will use bone marrow (BM) as a “glue” that is also easily accessible during the operative process by puncturing the patient’s iliac bone [16]. BM is rich in platelets and fibrinogen, which generates fibrin clots and is beneficial for tissue regeneration [17].

The development of injectable materials has focused on simulating the composition, morphology, and mechanical characteristics of natural cartilage extracellular matrix (ECM) [18]. Collagen type II, as the main component of hyaline cartilage ECM, is generally believed to have a crucial role in cartilage growth, proliferation, and the maintenance of the hyaline phenotype in cartilage [19–22]. The abilities mentioned above of collagen type II are attributed to its unique 3-dimensional protein structure [20] and particular peptide sequence [22]. Studies [23–25] have shown that compared with collagen type I scaffolds, scaffolds containing collagen type II are more conducive to maintaining the morphology and phenotype of chondrocytes and increasing the deposition of glycosaminoglycans (GAGs). However, no collagen type II product can be industrially produced in large quantities for clinical cartilage repair [24]. Reconstituted collagen type II is commonly used in existing research [25] but is costly and difficult to scale. Our previous research has prepared collagen type II scaffold materials with good mechanical properties and can achieve industrial mass production [26,27]. To accomplish the injectability of collagen type II in this study, collagen type II scaffolds were pulverized and used as the main body of the injectable material. Compared with other studies, the collagen type II content of these injectable materials is a qualitative leap.

To achieve in situ ACD repair under minimally invasive surgery, this study prepared injectable materials based on the collagen type II, BM acted as a glue, and thrombin (Th) acted as an additional supplement to shorten the formation time of fibrin clots and enhance the retention time of the injectable materials. For comparison, the F-gel was used as another glue formula. This article explored the properties of the injectable materials in vitro, verified the ACD repair and regeneration ability of the injectable materials in vivo, and explored the potential mechanisms. The successful completion of this project signified the advent of a groundbreaking era in minimally invasive, in situ cartilage repair, presenting a more convenient and less invasive treatment option for ACDs.

## Materials and Methods

### Prepare collagen type II-based injectable implanting material

Collagen type II scaffolds were prepared using established protocols [26]. In summary, first-generation porcine chondrocytes were combined with gelatin microspheres (150 to 180 μm in diameter) at 10^7^ cells/ml concentration. These cell-laden microspheres were then encapsulated in alginate hydrogels. Cavities were created within the hydrogel by dissolving the gel in an incubator set at 37 °C. The constructs were cultured in a suitable culture medium for 35 d. Following the initial culture period, the alginate was removed by incubating the constructs with a 55 mM sodium citrate solution. Once the alginate was dissolved, the alginate-free constructs were cultured for 10 d. Subsequently, decellularization was performed to obtain decellularized collagen type II scaffolds. Collagen type II powder is obtained through physical shearing and ultrasonic crushing (Fig. S1). The injectable materials consist of collagen type II powder (F-Col II) as the primary component, supplemented with additional components such as F-gel, BM, and Th. Three different formulations were employed to produce injectable materials based on collagen type II: (a) F-Col II with the addition of F-gel (F-Col II+F-gel), (b) F-Col II with the addition of BM (F-Col II+BM), and (c) F-Col II with the addition of BM and Th (F-Col II+BM+Th).

### Characterization of materials in vitro

#### Surface morphology characterization

The 3 injectable materials were immersed in ultrapure water and subsequently frozen at −20 °C. Freeze drying was performed before surface sputter coating. To secure the samples, the conductive tape was used to fix them onto sample holders. Surface morphology characterization was carried out using a scanning electron microscope (SEM).

#### Histology staining

The 3 injectable materials were dehydrated using sucrose and embedded in the optimal cutting temperature compound. The embedded materials were then sectioned to obtain 10-μm-thick slices using a cryostat (CryoStar NX70 cryostat, Thermo Scientific). Before staining, the sections were rinsed 3 times with phosphate-buffered saline (PBS). Then, standard protocols were employed, utilizing hematoxylin and eosin (H&E), Safranin-O (Saf-O), Masson’s trichrome staining techniques, and immunohistochemistry (IHC) staining.

#### Degradation in vitro

Injectable materials were prepared by individually weighing them and placing them into separate test tubes. One milliliter of pH 7.4 PBS buffer solution was added to each test tube. Weight loss experiments were conducted at 37 °C. The materials were retrieved from the test tubes, and filter paper was used to absorb the solution on the material surface. Subsequently, the materials were weighed.

Remaining mass rate (%) = *m_t_* / *m*_*0*_× 100%

The *m*_*0*_ represents the initial mass of the material in grams, and *m_t_* represents the mass of the material at time *t* in grams.

#### Mechanical testing

An Instron device (USA) equipped with an unconfined compression device was utilized. A constant compression load was applied to the 3 injectable materials at a rate of 200 μm per minute. The stress–strain curve obtained was used to determine the linear portion within the elastic region, enabling the calculation of the compression modulus of the materials.

#### Cytotoxicity

Human bone marrow mesenchymal stem cells (BMSCs) were expanded and cultured using MSC expansion medium to achieve stability. Using trypsin–EDTA (0.05%) to separate cells when they reach 70% confluence and transferring the cells to a centrifuge tube and centrifuge to obtain BMSC pellet.

1. Coculture: BMSCs were resuspended in MSC medium, and 4,000 cells were seeded in per 96-well plate well. After 5 h of culture, BMSCs adhered to the plate wall, and 3 injectable materials were placed in the culture medium for coculture. Fluid changes were performed daily.
2. Supernatant culture: The 3 injectable materials were immersed in MSC culture medium for 72 h at 37 °C. The supernatant from the injectable materials was collected and utilized to resuspend the BMSC pellet. Subsequently, BMSCs were seeded at a concentration of 4,000 cells in per 96-well plate well.

Cell Counting Kit-8 (CCK-8) assays were conducted at 12, 24, 48, and 72 h, with 3 replicate samples per group. The sample was washed with PBS, and a fresh culture medium (containing 10% CCK-8 solution) was added according to the CCK-8 assay protocol. The cells were incubated for 1 h in the incubator. Subsequently, the absorbance at 450 nm was measured.

### Animal surgery

**Fig. 1.**
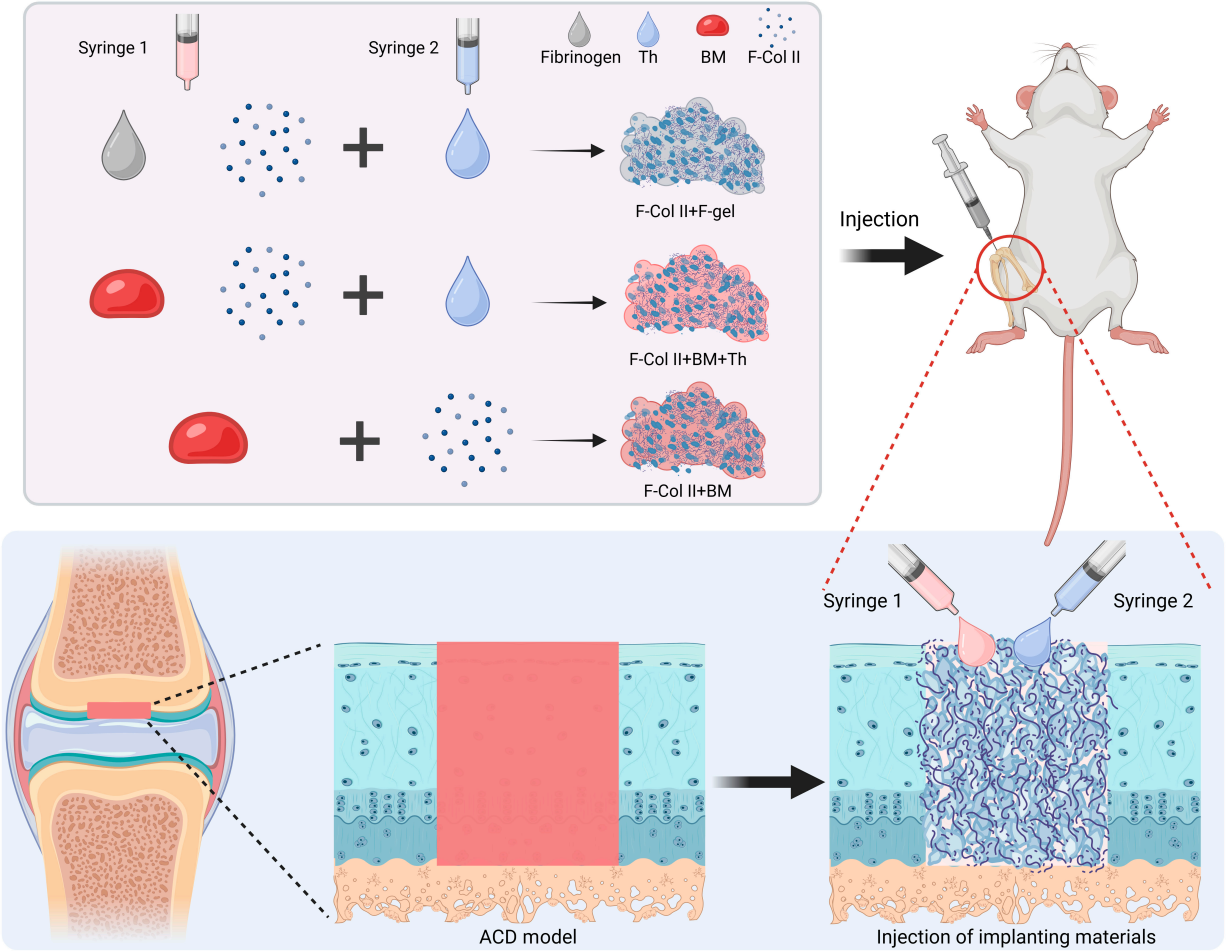
Preparation of injectable materials, ACD modeling, and injection of implanting materials.

The Animal Research Ethics Committee of the City University of Hong Kong has approved the animal experiments in this study (Approval number: AN-STA-00000025). Numerous preclinical and clinical experiments have shown that using BM or F-gel alone achieves fibrocartilage repair but not a hyaline cartilage phenotype. The untreated group can also achieve fibrocartilage repair in the rat cartilage defect model. Therefore, for animal ethics, to reduce the number of unnecessary experimental animals, this experiment did not include a relevant experimental group to test the separate use of BM or F-gel. A total of 21 male 8- to 9-week-old Sprague Dawley rats were used in this study (Table S1). The experimental design for the animal surgeries is shown in Fig. 1. In summary, 1.5-mm (critical size) [28,29] diameter ACDs were created in the hind limbs, and the modeling area extended deep into the subchondral bone plate, without involving the subchondral trabecular bone (no bleeding occurred during the surgical procedure). The BM was derived from the marrow cavity of the unmodeled lateral femur of rats. Then, 3 injectable materials were implanted. The Negative control group means modeling ACDs without material injection.

### Evaluation of repair and regeneration effects in vivo

#### Histology staining

Following the euthanasia of the rats, hind limbs were fixed in 4% paraformaldehyde for 48 h, and samples were subsequently decalcified in 10% EDTA for 14 d. Then, embedded the samples in paraffin and sectioned. For histological staining, standard protocols were employed, utilizing H&E, Saf-O, Masson’s trichrome staining techniques, Sirius Red, and IHC staining. Anti-collagen I antibody, anti-collagen II antibody, and anti-collagen VI antibody were used to label collagen type I (Col 1), collagen type II (Col 2), and collagen type VI (Col 6), respectively. We analyzed the Saf-O and IHC staining that could be quantitatively evaluated using ImageJ software (*n* = 3). Since the staining and photographic conditions of each photo are different, each image needs to be standardized to ensure the comparability of statistical data. When quantifying Saf-O staining, the nonspecific coloring degree of the bone layer was subtracted to standardize the data, and the coloring degree within the unit area of the cartilage around the repair area was defined as 1. In Col 1 staining, the nonspecific coloring degree of the cartilage layer was subtracted for data standardization, and the coloring degree per unit area of the bone layer was defined as 1. In Col 2 staining, the nonspecific coloring degree of the bone layer was subtracted for data standardization, and the coloration degree per unit area of the cartilage around the repair area was defined as 1. In Col 6 staining, the nonspecific coloring degree of the background was subtracted for data standardization, and the coloration degree per unit area of the cartilage around the repair area was defined as 1.

#### Biochemical assays and biomechanical testing

The region of interest was carefully extracted from the distal end of the fresh rat femur, followed by a 48-h digestion in papain solution (The tissue of Native group from the native cartilage adjacent to the defect area). Quantitative measurement of DNA was performed using the fluorescence Hoechst 33258 assay, GAG was performed using the 1,9-dimethyl methylene blue assay, and hydroxyproline was performed using the hydroxyproline assay. Immediately after obtaining the distal femur, biomechanical testing was conducted. The Instron apparatus with a compression device (USA) was utilized to analyze Young’s modulus. A compressive load was applied to the region of regeneration tissue.

#### Micro-CT scan

The hind limbs were immediately scanned using the Scanco Xtreme II micro-computed tomography (CT) scanner (the tissue of Native group from the native cartilage adjacent to the defect area). Mimics and CTAn software were employed for image 3D reconstruction and bone morphometric analysis. The regions of the surgical site were selected as interest areas, and the subchondral cortical bone and trabecular bone were separated. Microstructural parameters of trabecular bone included trabecular separation (Tb.Sp), trabecular thickness (Tb.Th), bone volume fraction (BV/TV), and trabecular number (Tb.N), while cortical bone morphometric parameters included average cortical thickness (Ct.Th) and cortical area fraction (Ct.Ar/Tt.Ar).

#### RNA sequencing

The rats were euthanized to conduct RNA sequencing. Regenerated cartilage tissues from the surgical site on the 100th day, as well as native cartilage adjacent to the defect area (nonsurgical areas), were collected, with 3 samples per group, and rapidly frozen in liquid nitrogen. BGI Tech Solutions Co. Ltd was responsible for RNA extraction, library construction, and sequencing. Intergroup statistical analysis was conducted using DESeq2, considering genes to be differentially expressed when the *P* < 0.05 and the absolute value of log2 fold change > 1. Gene Ontology (GO) analysis, Gene Set Enrichment Analysis (GSEA), and Kyoto Encyclopedia of Genes and Genomes (KEGG) analysis were employed to identify the biological processes and pathways.

### Statistics analysis

The staining results were analyzed using ImageJ software. All data were analyzed using GraphPad Prism 9 (California, USA) and presented as mean ± standard deviation. One-way or 2-way analysis of variance (ANOVA) was performed to assess differences between groups or multiple data sets. The significance level was set at *P* < 0.05. In the results, * denotes *P* < 0.05, ** denotes *P* < 0.01, *** denotes *P* < 0.001, and **** denotes *P* < 0.0001.

## Results

### Characterization of materials in vitro

**Fig. 2.**
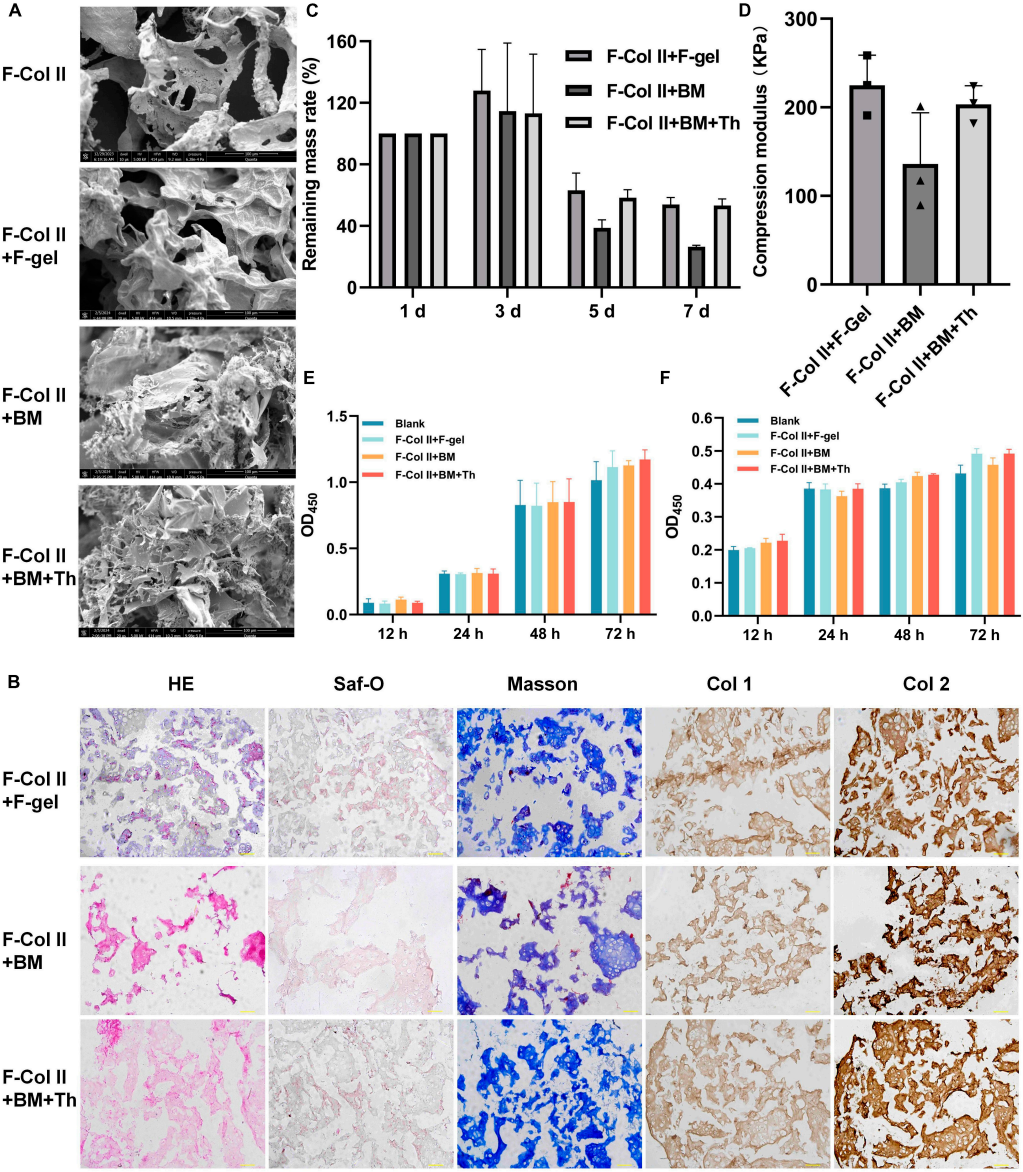
The characterization of injectable materials in vitro. (A) SEM of the F-Col II and injectable materials (image magnified 500 times; the scale bar in the images represents 100 μm). (B) H&E, Saf-O, Masson staining, and IHC staining for Col 1 and Col 2. The scale bar in the images represents 200 μm. (C) The degradation experiment of the injectable materials under 37 °C in PBS. (D) Mechanical testing analysis for compression modulus (at the 20% deformation of the injectable materials). (E) The CCK-8 analysis of the coculture with injectable materials and (F) culture with the supernatant of injectable materials.

The surface morphology of F-Col II, F-Col II+F-gel, F-Col II+BM, and F-Col II+BM+Th was assessed using SEM. As depicted in Fig. 2A, F-Col II exhibited a porous and loosely structured appearance. Compared with the smooth surface of the F-Col II+F-gel, the surface of the F-Col II+BM and F-Col II+BM+Th is rougher, indicating the presence of more components in BM. The frozen sections (Fig. 2B) of the 3 injectable materials revealed a significant decrease in the proportion of F-Col II in F-Col II+BM compared to the other 2 groups, which indicates that the formulation with only BM has a poorer ability to bind F-Col than the other 3 formulations. Saf-O staining results indicate the presence of a small amount of GAG in all 3 materials. Additionally, Masson’s staining results confirm the composition of the materials as collagen. By observing the positive results for Col 2, it can be concluded that these 3 injectable materials primarily consist of collagen type II, and the weak positivity for Col 1 may be attributed to the adhesive component.

The in vitro degradation results of the injectable material showed that on the seventh day, the remaining mass rates (%) of the F-Col II+BM+Th group and the F-Col II+F-gel group were similar, 53.286 ± 4.228 and 53.901 ± 4.615, respectively. The F-Col II+BM group showed less mass retention, 26.481 ± 0.902 (Fig. 2C). F-Col II+BM+Th exhibited a compression modulus (Fig. 2D) of 203.5 ± 20.90 kPa, which was similar to F-Col II+F-gel (224.90 ± 33.95 kPa). F-Col II+BM, which had the lowest compression modulus, reached 136.0 ± 57.96 kPa. The CCK-8 results (Fig. 2E and F) indicate that the 3 injectable materials have no cytotoxicity, have good biocompatibility, and can be used for in vivo experiments.

### Evaluation of repair and regeneration effects in vivo

#### Histology and IHC staining

**Fig. 3.**
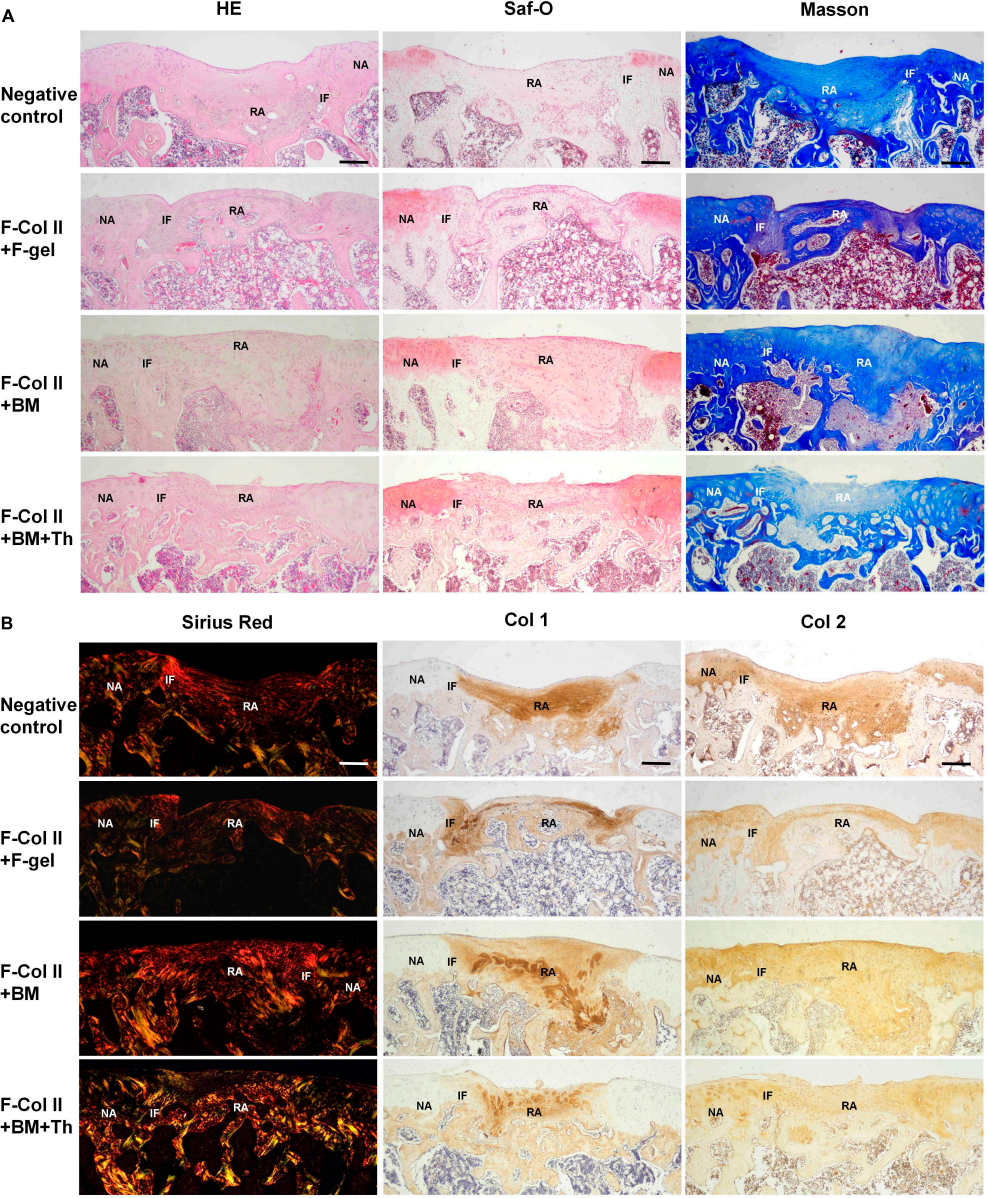
Histological staining of ACDs was performed on day 50 after material injection. (A) H&E, Saf-O, and Masson staining. (B) Sirius Red staining and IHC staining for Col 1 and Col 2. The scale bar in the images represents 200 μm. Interface (IF), regeneration area (RA), and native area (NA).

In the 50-d staining (Fig. 3), all groups exhibited satisfactory fusion between the regenerated tissue and adjacent tissue. However, F-Col II+F-gel showed a slight deficiency in filling the interface between the regeneration area and the native area, which may be attributed to the metabolic effects of F-gel within the body. F-Col II+BM and F-Col II+BM+Th achieved excellent filling of ACDs. Regarding Saf-O staining, no GAG deposition was observed in the filling area compared to normal cartilage at 50 d postimplantation. The Masson staining showed the collagen deposition. In Sirius Red staining, collagen type II appears green under polarized light, and collagen type I appears red or yellow. The results showed that except for the F-Col II+BM+Th cartilage layer, which was partially green, the other groups were all red. Comparing the IHC staining of Col 1 and Col 2 (where dark brown indicates positive and light yellow/white indicates negative), it was observed that at 50 d postrepair, the regenerated tissue displayed a mixed phenotype, characterized by the simultaneous expression of both collagen type I and type II.

**Fig. 4.**
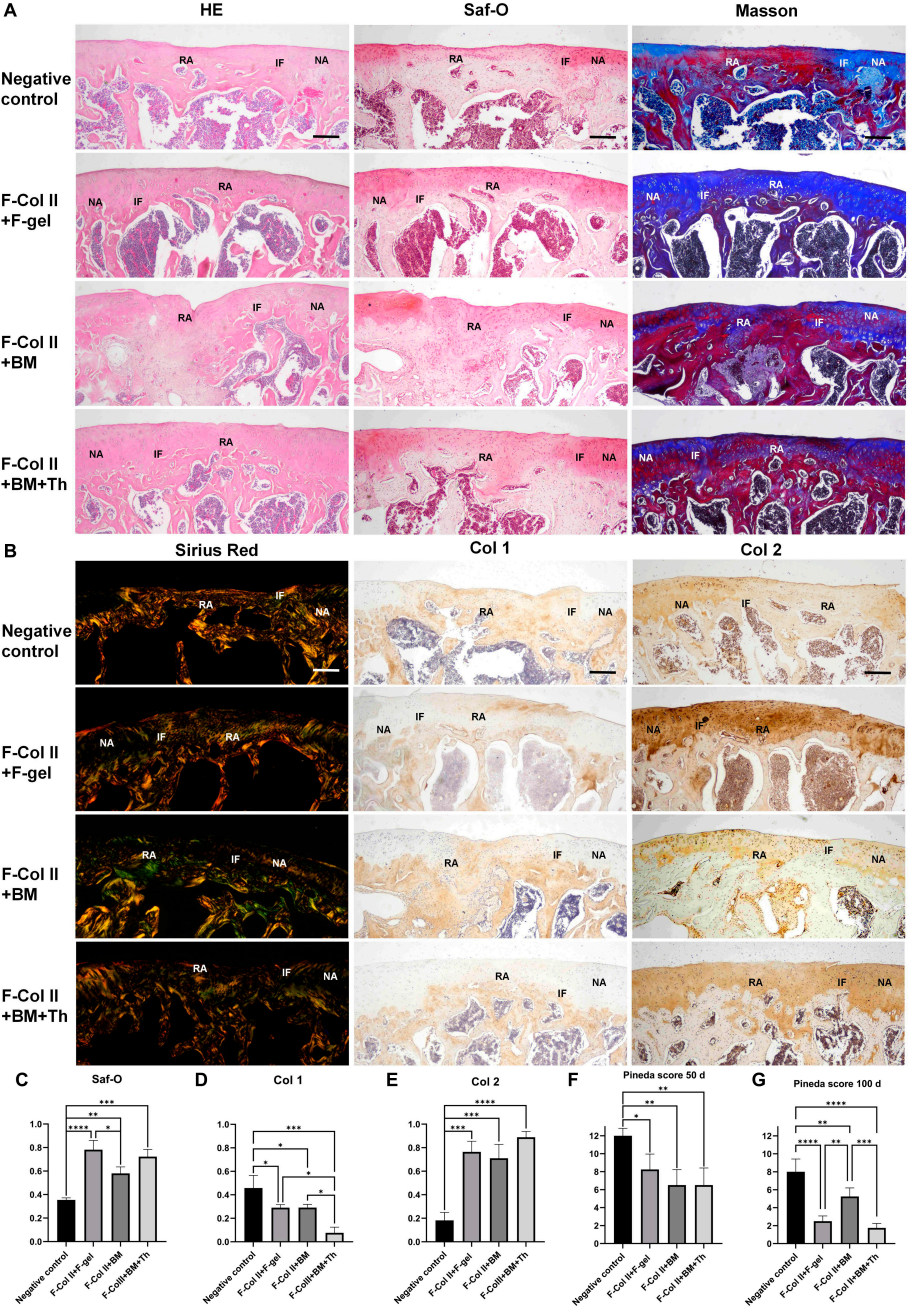
Histological staining of ACDs was performed on day 100 after material injection. (A) H&E, Saf-O, and Masson staining. (B) Sirius Red staining and IHC staining for Col 1 and Col 2. The scale bar in the images represents 200 μm. Interface (IF), regeneration area (RA), and native area (NA). (C to E) The Saf-O and IHC staining (Col 1 and Col 2) were quantitatively evaluated using ImageJ software (*n* = 3). (F and G) Pineda scores of each group 50 and 100 d after surgery (four orthopedic surgeons not related to this study were invited to score based on the staining results).

In the 100-d H&E staining (Fig. 4A), F-Col II+BM displayed uneven surface depressions in the regenerated area, and the regenerated tissue did not demonstrate the characteristic 4-layer structure of cartilage. This outcome could be attributed to the limited ability of BM to provide adhesion for F-Col II, resulting in material detachment after implantation and adversely affecting the repair outcome. On the other hand, F-Col II+F-gel and F-Col II+BM+Th exhibited favorable cartilage repair effects, with smooth transitions at the interface and regenerated areas displaying a 4-layer structure resembling native cartilage. Except for F-Col II+BM, distinct demarcations between cartilage and bone tissues were observed in the remaining groups, characterized by the presence of tidemarks. Compared to F-Col II+BM and F-Col II+BM+Th, F-Col II+F-gel exhibited significantly lower subchondral bone volume and wider gaps between trabeculae, indicating a slightly inferior capacity for subchondral bone regeneration. Regarding Saf-O and Masson staining, at 100 d postimplantation, all injectable material groups exhibited GAG and collagen deposition in the cartilage layer surpassing the Negative control group. Notably, F-Col II+F-gel and F-Col II+BM+Th displayed more pronounced GAG staining and higher GAG content in comparison to F-Col II+BM, indicating superior regenerative effects. In Sirius Red staining results (Fig. 4B), except for the Negative control group, the regenerated cartilage in all other groups showed “green”, representing collagen type II. However, it can be observed that the arrangement of collagen type II fibers is different from that of surrounding native cartilage. The IHC staining of Col 1 and Col 2 at 100 d revealed that the collagen phenotype in F-Col II+BM+Th had become consistent with the surrounding native tissue. Specifically, the cartilage layer exhibited positive staining for collagen type II, while the bone layer exhibited positive staining for collagen type I. In contrast, although there had been a qualitative change compared to the 50-d results, some of the regenerated tissue in F-Col II+F-gel and F-Col II+BM still exhibited a mixed phenotype. The Saf-O and IHC staining were quantitatively evaluated using ImageJ software (*n* = 3; Fig. 4C to E). The Saf-O coloration of the surrounding native cartilage was used as a reference, with a value of 1. The results indicated that the coloration of the regenerated tissue in the F-Col II+BM group was superior to that of the Negative control group but inferior to that of the F-Col II+F-gel group. However, there was no significant difference in coloration between the F-Col II+F-gel group and the F-Col II+BM+Th group. For Col 1 staining, the coloration of the surrounding native bone was used as the reference, with a value of 1. The regenerated tissue in both the F-Col II+F-gel group and F-Col II+BM group exhibited significantly lower collagen type I content than the Negative control group. However, the collagen type I content in these 2 groups was significantly higher than in the F-Col II+BM+Th group. Regarding the Col 2 staining, the coloration of the surrounding native cartilage was used as the reference, with a value of 1. The Negative control group had significantly lower collagen type II content in the regenerated tissue compared to each injectable scaffold group. Among the injectable scaffold groups, the F-Col II+BM+Th group demonstrated the highest collagen type II content. Moreover, the Pineda score (Fig. 4F and G) was consistent with the above findings. These results suggest that the F-Col II+BM+Th has a superior impact on cartilage regeneration compared to other experimental groups (Table S2 for scoring criteria).

**Fig. 5.**
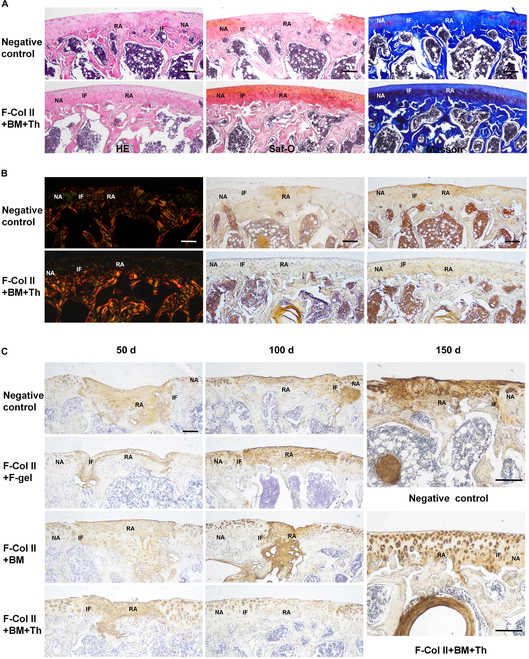
Histological staining of ACDs. (A) H&E, Saf-O, and Masson staining, and (B) Sirius Red staining and IHC staining for Col 1 and Col 2 on day 150 after material injection. (C) The IHC staining for Col 6 on days 50, 100, and 150 after material injection. The scale bar in the images represents 200 μm. Interface (IF), regeneration area (RA), and native area (NA).

After 150 d of repair and regeneration (Fig. 5A), compared with the Negative control group, the regenerated cartilage in the F-Col II+BM+Th group had been perfectly integrated with the surrounding normal cartilage. The thickness and morphology of cartilage, as well as the deposition of GAG and collagen, tend to be consistent with natural cartilage. Compared with the Negative control group, the arrangement of collagen type II fibers of regenerated cartilage in the F-Col II+BM+Th group was consistent with the surrounding native cartilage in Sirius Red staining. The IHC staining showed the regenerated cartilage in the F-Col II+BM+Th group maintained the hyaline cartilage phenotype after 150 d (Fig. 5B).

Under physiological conditions, Col 6 is an anchor between chondrocytes and the pericellular matrix in articular cartilage. It is usually only distributed around the periphery of chondrocytes. Col 6 will diffusely distribute throughout the ECM when inflammation occurs in the articular cartilage [30,31]. The results in Fig. 5C show that in the 50-d staining results, the cartilage defect sites in each group were all positive for Col 6, indicating that in the early stage of cartilage repair, the repair area was an inflammatory environment. In the 100-d staining results, the Col 6 staining of the regenerated cartilage in the cartilage repair area of ​​the F-Col II+BM+Th group was similar to the surrounding normal cartilage, indicating that the inflammation in the cartilage defect site of the F-Col II+BM+Th group had subsided. The ECMs of cartilage defect sites in other groups were still Col 6 positive, indicating that the inflammatory environment still existed. In the quantitative evaluation, the COL6 content of the surrounding normal cartilage is defined as 1, and the closer to 1, the better the degree of repair. Values greater than 1 indicate abnormal COL6 content and distribution (Fig. S2D). In the 150-d staining results, the cartilage defect area in the Negative control group was still strongly positive for Col 6, indicating that the local area was still an inflammatory environment. The regenerated cartilage in the cartilage repair area of ​​the F-Col II+BM+Th group was consistent with the surrounding normal cartilage. See Fig. S2 for high-resolution magnified images.

#### Biochemical and mechanical analyses

**Fig. 6.**
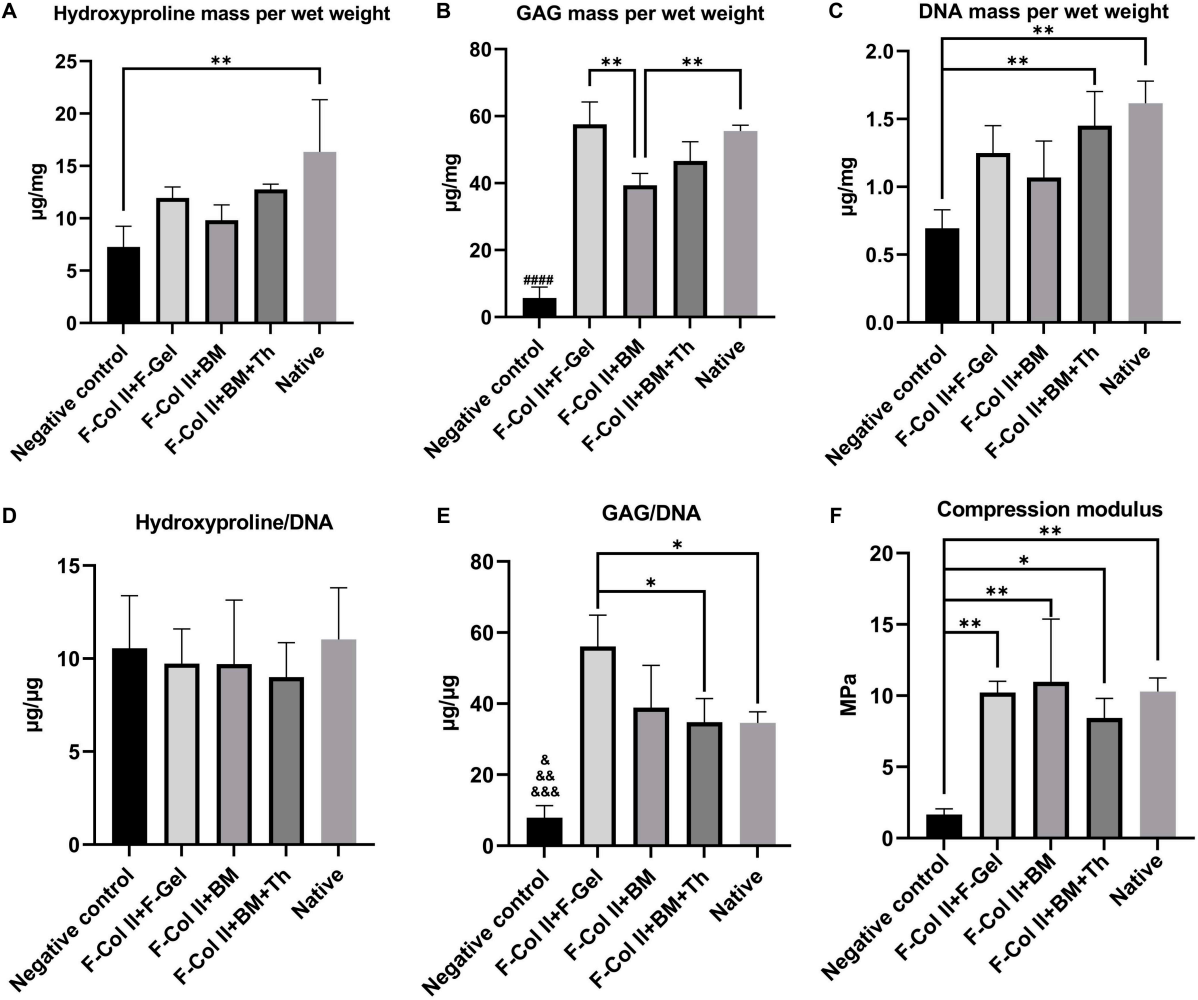
Biochemical and mechanical analyses of regeneration areas on day 100 after modeling and implantation. (A to C) The quantification results of hydroxyproline, GAGs (^####^, *P* < 0.0001 Negative control group versus all other groups), and DNA for regenerated areas. (D and E) The results of hydroxyproline/DNA and GAGs/DNA (^&^, *P* < 0.05 Negative control group versus Native group; ^&&^, *P* < 0.01 Negative control versus F-Col II+BM and F-Col II+BM+Th; ^&&&^, *P* < 0.001 Negative control group versus F-Col II+F-gel) for regenerated areas. (F) Biomechanical analysis for regenerated areas (osteochondral, confined).

The biochemical and morphological staining results of the regeneration area after 100 d of implantation with the 3 injectable materials demonstrate a convergence. As shown in Fig. 6A, the hydroxyproline content in 3 injectable materials is higher than that in the Negative control group, indicating a substantial accumulation of collagen, and no significant differences among the 3 materials. Figure 6B illustrates that the deposition of GAG in the regeneration area is significantly enhanced in the 3 material groups compared to the Negative control group (*P* < 0.0001). F-Col II+F-gel and the Native group displayed significantly better collagen deposition than F-Col II+BM (*P* < 0.01). Figure 6C and D represents the DNA mass and hydroxyproline/DNA ratio, in the regeneration area of the 3 injectable materials, no significant differences were observed, indicating comparable levels of collagen content produced by individual cells. However, the GAG/DNA ratio (Fig. 6E) indicated that in the F-Col II+F-gel group, the levels of GAG content produced by individual cells were higher than in F-Col II+BM+Th and Native groups (*P* < 0.05). The statistical analysis of the stress–strain curves in the regeneration area (*n* = 3), as presented in Fig. 6F, revealed a significantly higher compression modulus of the regenerated tissue in the 3 injectable materials compared to the Negative control (*P* < 0.05 and *P* < 0.01). However, among the 3 different repair materials, no significant differences were observed, and their compression modulus was similar to that of the surrounding native tissues (about 10 MPa).

#### Subchondral bone under micro-CT

**Fig. 7.**
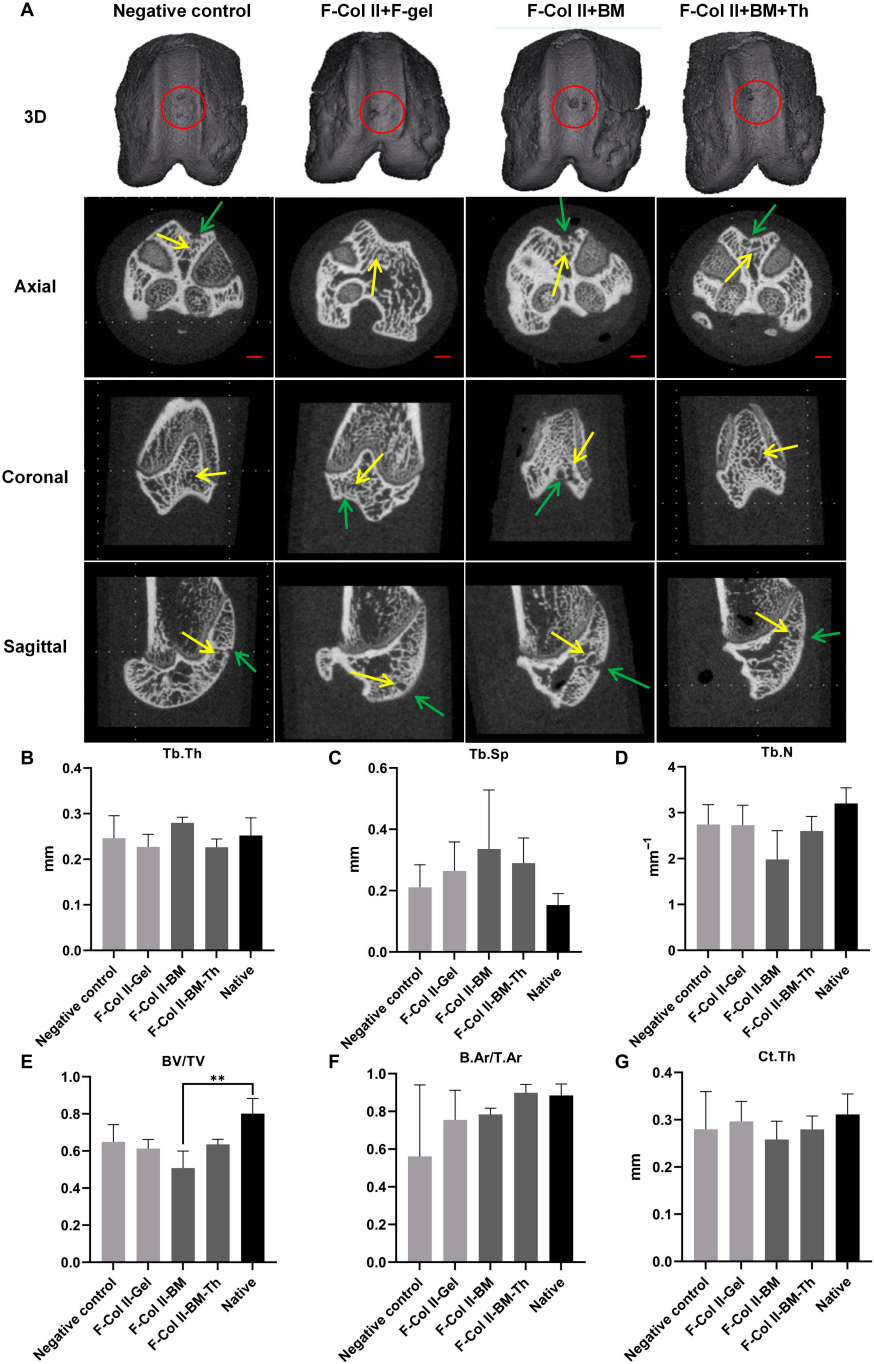
The subchondral bone under micro-CT of regeneration areas on day 100 after material injection. (A) 3D reconstruction model and images of the ACD area in 3 axes. (The red circle is the area of interest below the regenerated tissue. The green arrow points to the subchondral bone plate, and the yellow arrow points to the subchondral cancellous bone.) Scale bar: 1 mm. (B to E) The analysis results of Tb.Th, Tb.Sp, Tb.N, and BV/TV. (F and G) Ct.Ar/Tt.Ar and Ct.Th.

Micro-CT analysis further elucidated the degree of influence on the subchondral bone with injectable materials for ACDs. The red circles denote the regions of interest, the green arrows indicate the subchondral bone plate, and the yellow arrows highlight the trabecular bone (Fig. 7A). Statistical analysis (*n* = 3) of the regions of interest revealed no statistically significant differences between materials groups and the native cartilage adjacent to the defect area) in Tb.Th (Fig. 7B) and Ct.Th (Fig. 7G).

Tb.N values in F-Col II+BM were lower compared to the other groups, while the Tb.Sp values were higher (with no statistically significant difference observed) (Fig. 7C and D). These findings suggest that F-Col II+BM influences the number of bone trabeculae under the regeneration cartilage area, accompanied by larger gaps between the trabeculae. The BV/TV values (Fig. 7E) in F-Col II+BM were significantly lower than surrounding native tissues (*P* < 0.01). Regarding the B.Ar/T.Ar (Fig. 7F), among the various materials and native tissue, no statistically significant differences in the subchondral bone plate, and all groups exhibited slightly better results than the Negative control group.

#### Transcriptome analysis

Gene expression analysis was conducted on samples of Gene expression analysis of the Regenerated cartilage in the defect area (RC-D) and Native cartilage adjacent to the defect area (NC-AD) of F-Col II+BM+Th group (*n* = 3), resulting in the identification of a total of 24,608 genes. A total of 3,896 genes were up-regulated, while 2,498 genes were down-regulated. Principal component analysis revealed a notable separation between the 2 sample groups (Fig. S3).

**Fig. 8.**
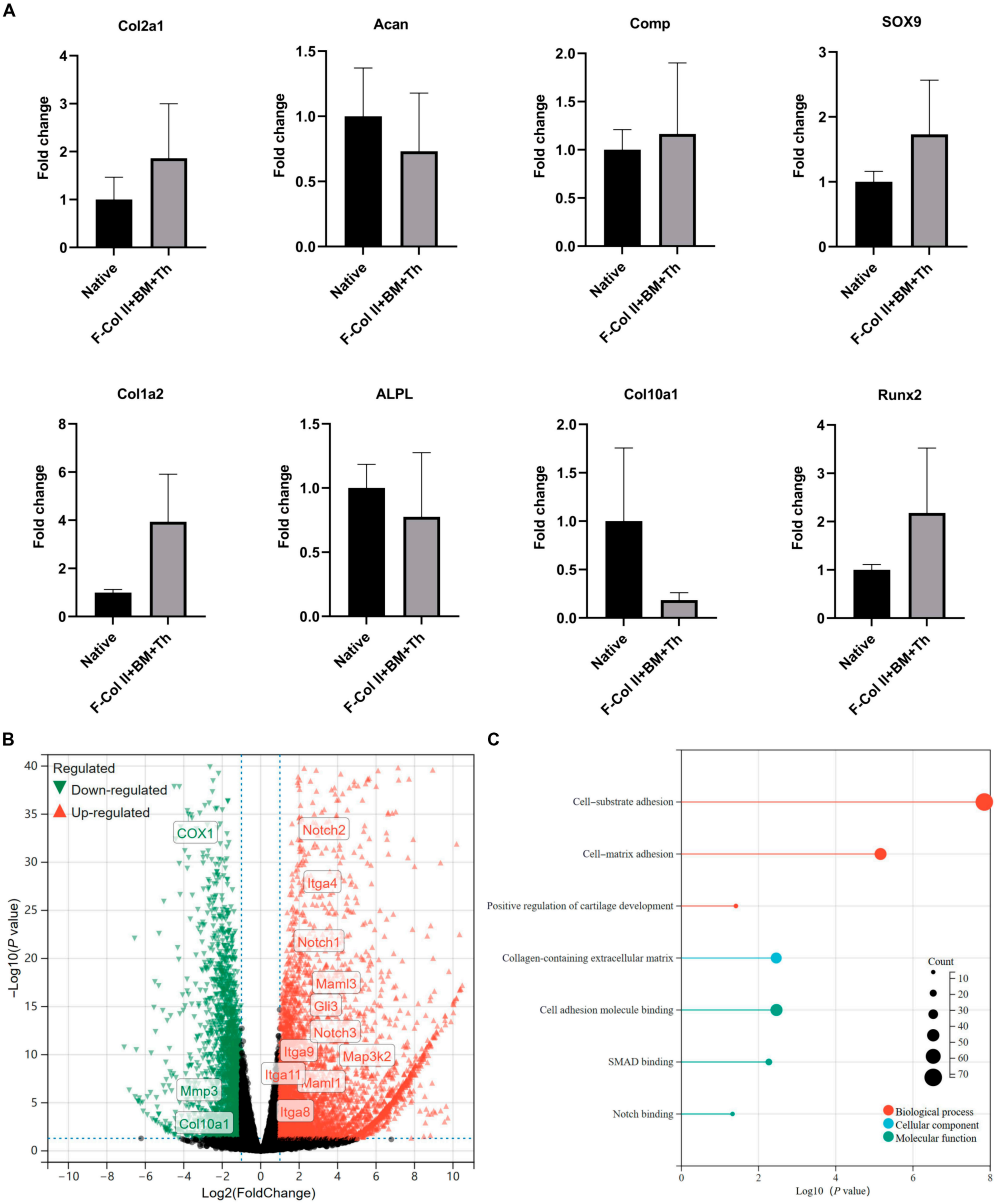
Gene expression analysis of the RC-D versus NC-AD of F-Col II+BM+Th group on day 100. (A) Relative expression of essential genes. (B) Volcano plot of the genes. (C) Gene enrichment analysis by GO.

When investigating specific gene expression levels with NC-AD as the reference (Fig. 8A), the RC-D of F-Col II-BM-Th exhibited higher expression levels of Col2a1, Sox9, Col1a2, Comp (cartilage oligomeric matrix protein), and Runx2 genes. Simultaneously, genes such as Acan (aggrecan), Alpl (alkaline phosphatase), and Col10a1 demonstrated relatively lower expression levels. A volcano plot was generated to visualize the expression of genes (Fig. 8B), with an emphasis on genes associated with cartilage tissue. The results showed reduced expression of cartilage degradation-related genes, such as MMP13 (matrix metalloproteinase), as well as genes associated with OA, including COX1. At the same time, the essential genes involved in the Notch pathway were up-regulated, such as Notch1, Notch2, Notch3, and Maml1,3. The up-regulation of Itga4 (integrin subunit alpha 4), Itga8, Itga9, and Itga11 was showed in the volcano plot, and up-regulation of Itga has been shown to contribute to cartilage repair and regeneration [32]. GO analysis (Fig. 8C) was conducted to assess the interaction of the differentially expressed gene (DEG) between the RC-D and NC-AD of the F-Col II+BM+Th group. The results indicate the up-regulation of processes related to “positive regulation of cartilage development”, “collagen-containing extracellular matrix”, “cell-substrate adhesion”, and “cell-matrix adhesion” (Fig. S4). These findings suggest that F-Col II+BM+Th creates a favorable microenvironment for cell adhesion, intercellular communication, and collagen synthesis, which aligns with the observations from IHC staining and biochemical testing.

**Fig. 9.**
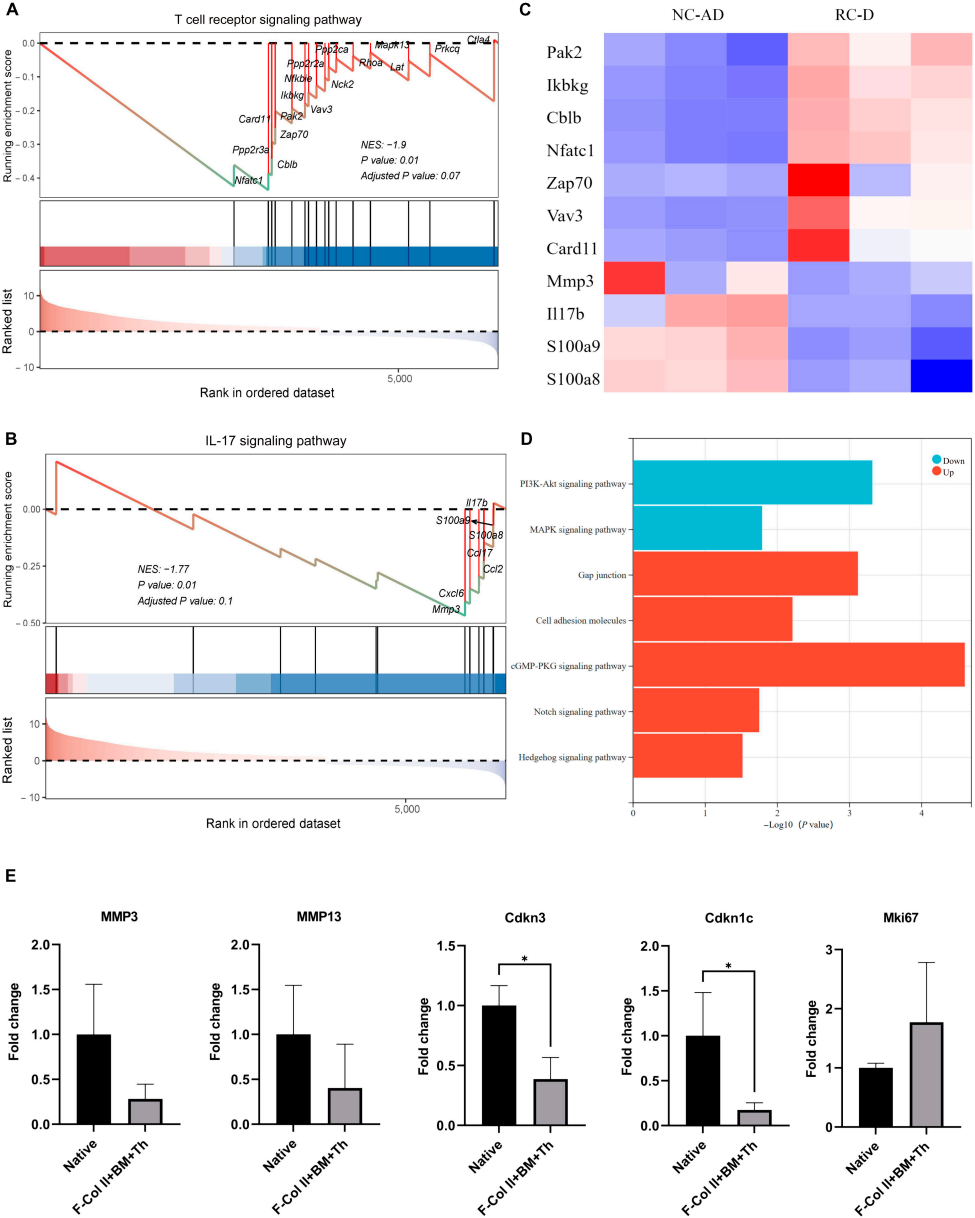
Gene expression analysis of the RC-D versus NC-AD of F-Col II+BM+Th group on day 100. (A) GSEA of TCR signaling pathway. (B) GSEA of IL-17 signaling pathway. (C) Heatmap of typical DEGs associated with TCR and IL-17 signaling pathway. (D) Gene enrichment analysis by KEGG. (E) The expression of MMP3, MMP13, Cdkn3, Cdkn1c, and Mki67 genes.

In addition, through the GSEA (Fig. 9A and B), the process was verified that F-Col II+BM+Th can control immunity by inhibiting the T cell receptor (TCR) signaling pathway (normalized enrichment scores [NES] = −1.9 *P* value = 0.01) and interleukin-17 (IL-17) signaling pathway (NES = −1.77 *P* value = 0.01), which will decrease cartilage degradation [33,34]. Heatmap (Fig. 9C) of typical DEGs associated with TCR signaling pathway and IL-17 signaling pathway. The critical genes of the TCR signaling pathway were up-regulated, such as Casitas B-lineage lymphoma b (Cblb), zeta-chain-associated protein kinase 70 (Zap70), and nuclear factor of activated T cells 1 (Nfatc1). In the TCR signaling pathway, Cblb functions as a negative regulator, mainly inhibiting signaling by promoting the ubiquitination of critical proteins, such as Zap70 [35]. The down-regulation of IL-17 signaling pathway-related genes MMP3 and IL-17b also proves the inhibition of the pathway. Furthermore, KEGG analysis (Fig. 9D) revealed that F-Col II+BM+Th may regulate cartilage repair and regeneration by various pathways, such as the phosphoinositide kinase-protein kinase B (PI3K-Akt) pathway (NES = −0.7306), mitogen-activated protein kinase (MAPK) pathway (NES = −1.2212), cyclic guanosine monophosphate-protein kinase G (cGMP-PKG) pathway (NES = 0.9376), Hedgehog pathway (NES = 0.6771), and Notch pathway (NES = 0.7701). In addition, RNA sequencing results also showed that the expression of MMP3, MMP13, Cdkn3, and Cdkn1c in RC-D was down-regulated compared with NC-AD (Fig. 9E). MMP3 and MMP13 are arthritis-related genes, while Cdkn genes that negatively regulate cell proliferation and are often considered to be related to cell senescence [33,36,37]. The down-regulation of Cdkn3 and Cdkn1c genes in RC-D was statistically significant (*P* value < 0.05). In addition, the Mki67 gene, an indicator commonly used to evaluate cell proliferation in RC-D, was up-regulated compared with NC-AD (Fig. 9E), which also suggests that chondrocytes in the regenerated tissue proliferated vigorously.

## Discussion

ACDs will develop into OA if not treated in time. However, with existing cartilage treatment methods, the regenerated tissue is usually fibrocartilage [5]. Fibrocartilage lacks normal biological functions of resistance to pressure and wear, so its presence causes further cartilage degeneration. This highlights the urgency and societal value of achieving hyaline articular cartilage regeneration in ACDs. The development of injectable materials allows for minimally invasive repair of ACDs [13]. However, balancing the injectability of the material with good articular cartilage repair ability is a challenge. Solving this dilemma requires consideration of the selection of 2 essential components of the injectable material: the binder and the host material. The first is the choice of adhesive, which must consider biocompatibility and biodegradability, which makes natural substances a good option. BM has the feasibility of clinical transformation and the convenience of clinical operation. BM aspiration clots have become a common cartilage repair material [16]. The second is the selection of the primary material. In the ECM of articular cartilage, collagen type II, as one of the most critical components, has been proven to play an indispensable role in the repair and regeneration of articular cartilage and the maintenance of hyaline phenotype [22,24,25]. This study selected collagen type II as the central part of the injectable material. Although there are studies [24,25,38] on injectable collagen type II hydrogels, the content of collagen type II in the hydrogels is relatively low.

The findings of this study once again demonstrate the significant role of collagen type II in cartilage repair and regeneration. The staining results obtained 100 d after surgery demonstrate that compared to the negative control group, all injectable collagen type II scaffold groups exhibit increased deposition of GAGs and collagen. The Sirius Red staining results reveal that the repair area in the negative control group appears similar in color to the subchondral bone, exhibiting a yellow-red hue. In contrast, the repair areas in all injectable collagen type II scaffold groups exhibit a color consistent with the surrounding normal cartilage. The IHC analysis indicates that, although the repair tissue in the negative control group has reached a level flush with the surrounding cartilage, the thickness of the cartilage layer is thin and exhibits characteristics of fibrous tissue (positive for Col 1 and negative for Col 2), such repair is prone to gradual degradation. In contrast, all injectable collagen type II scaffold groups show strong positive staining for Col 2, negative staining for Col 1, or partial weak positive staining for Col 1. These results suggest that the regenerated tissue predominantly exhibits a hyaline cartilage phenotype and possesses the functional properties of normal articular hyaline cartilage.

Adhesives, as an integral component of injectable materials, play a crucial role in the repair process. In this experiment, 3 different adhesive formulations were utilized. In vitro experiments revealed that the addition of BM to collagen type II resulted in the attachment of active ingredients on its surface, not in conventional medical glues like fibrin glue. Compared with BM, BM combine Th exhibited superior adhesion to F-Col II, as evidenced by a lower degradation rate and higher compression modulus. The mechanical properties and degradation rates of injectable collagen type II materials prepared with BM combine Th and F-gel were found to be comparable. Observations from the 50-d staining sections showed a gully between the F-Col II-F-gel group and surrounding native cartilage, potentially due to the rapid degradation of F-gel in vivo. However, this phenomenon was not observed in the F-Col II+BM and F-Col II+BM+Th groups. Sirius Red staining results of the F-Col II+BM group revealed a red color in the upper layer of the repair area, consistent with the defect area of the negative control. Notably, no repair material was observed in the upper layer of the repair area in the H&E staining of the F-Col II+BM group, indicating that BM exhibited poor bonding ability to F-Col II and was prone to degradation and detachment after implantation, which causes fibrous tissue filling. Conversely, the addition of BM combine Th improved the bonding ability, aligning with the in vitro results. Staining results at 100 d demonstrated that the easy degradation of F-gel and the low adhesion of BM negatively impacted cartilage repair efficacy. IHC revealed local weak positivity for Col 1, suggesting the presence of nonhyaline cartilage repair in the ACD area. Furthermore, Saf-O staining and biochemical detection concurrently indicated low deposition of GAGs in the F-Col II+BM group. In contrast, collagen type II scaffolds with BM combine Th as the adhesive demonstrated a favorable hyaline cartilage repair phenotype. Combining the above results to analyze the IHC staining results of Col 6, the cartilage defect site in the Negative control group was in an inflammatory environment in the early stage, resulting in the inability of the proliferating fibrocartilage to fill the defect. At 100 d, although the defect site had been filled with fibrocartilage, the fibrocartilage was not hyaline cartilage, which led to the continuation of inflammation. Even if the repair was done for 150 d, this nonhyaline cartilage fibrous repair maintained the local inflammatory environment. Even in the F-Col II+F-gel group and F-Col II+BM group that achieved like-hyaline cartilage repair, this inflammatory environment still existed at 100 d. In contrast, only the F-Col II+BM+Th group that achieved hyaline cartilage repair showed staining results similar to the surrounding normal cartilage at 100 d, indicating the subsidence of local inflammation maintained at 150 d. The above results fully prove that without any intervention, the cartilage defect site will continue to keep an inflammatory state, which will inhibit cartilage repair (even fibrocartilage repair), and poor repair will cause local inflammation to persist, forming a vicious cycle, which is consistent with many research theories [33,39,40]. The F-Col II+BM+Th group seems to have achieved good repair and reduced inflammation, and the subsided inflammation is conducive to the repair and maintenance of hyaline cartilage.

In this study, we did not verify the repair properties of BM and Th other than adhesion. However, the related study [17] has shown that BM contains many active ingredients that can form fibrous clots, which benefit tissue repair and regeneration. According to the report in [41] the cartilage repair process, the proliferative phase begins at 1.5 months, the remodeling phase starts at 3 to 6 months, and the fibrous clots will degrade within 1 month. Therefore, in the proliferative and remodeling phases, Col II is the main component assisting hyaline cartilage regeneration. Consequently, we believe that BM+Th may be helpful in the early stages of repair, but in the middle and late stages of repair, it is mainly Col II that plays a role.

Based on the excellent hyaline cartilage repair ability of F-Col II+BM+Th, RNA sequencing analysis was conducted on the regeneration area and surrounding native articular cartilage to verify how close or far apart the two are. Because the ultimate goal is to keep the regeneration area cartilage consistent with the surrounding native articular cartilage. Transcriptome analysis demonstrated that genes related to cell adhesion and collagen-containing ECM were up-regulated, and pathways related to chondrogenesis were regulated. Such as the up-regulation of the Notch signaling pathway, according to research reports, the Notch signaling pathway plays a crucial role [42,43] in articular cartilage formation, phenotype maintenance, and degeneration. Existing studies [42,44] believe that early activation of the Notch signaling pathway is necessary for hyaline cartilage formation. cGMP-PKG and PI3K-Akt signaling pathways play a role in regulating cell proliferation, differentiation, and metabolism in cartilage. It affects multiple signaling pathways and gene expression within cells, thereby regulating chondrocyte function. Studies have shown that MAPK [45,46] is an essential upstream signaling pathway in proteolytic cartilage degradation, which can lead to the up-regulation of MMP expression and lead to OA. In this study, F-Col II+BM+Th inhibits the MAPK signaling pathway and down-regulation the lever of MMP13 and MMP3, benefiting hyaline cartilage. The Hedgehog signaling pathway48 plays an essential role in adult cartilage maintenance. It regulates the proliferation and differentiation of chondrocytes and the maintenance and repair of articular cartilage.

The inflammatory environment resulting from cartilage damage poses a significant obstacle to the repair, regeneration, and maintenance of a hyaline phenotype in ACDs. Under inflammatory conditions, chondrocytes undergo dedifferentiation, becoming a fibroblast-like phenotype, which promotes the formation of fibrocartilage during the repair process [40]. Upon cartilage injury, neutrophils accumulate in large numbers, secreting proinflammatory mediators and recruiting macrophages, dendritic cells (DCs), and T cells. T helper 1 (Th1) cells polarize macrophages into M1 macrophages, further releasing proinflammatory factors [40]. DCs activate Th1 and Th17 cells, thereby contributing to cartilage degeneration [33]. Conversely, macrophages can be polarized during the repair phase into M2 macrophages through IL-4 action secreted by Th2 cells. M2 macrophages exhibit anti-inflammatory properties and secrete factors promoting chondroblast activity, inhibiting the inflammatory response and facilitating cartilage repair [39]. Additionally, DCs promote the chondrogenic differentiation of MSCs and induce the proliferation of regulatory T cells by secreting IL-10 [47]. Regulatory T cells, in turn, promote the expression of IL-10 and transforming growth factor beta 1, which help inhibit inflammation and facilitate cartilage formation. Therefore, reversing the local inflammatory environment in ACDs is particularly important for the successful regeneration of hyaline cartilage. The activation of the TCR signaling pathway and IL-17 signaling pathway can promote inflammation, causing the degradation of articular cartilage and the occurrence of OA [33]. The inhibition of the TCR signaling pathway and IL-17 signaling pathway proves that injectable materials may regulate immunity to ensure that the defect area is in a noninflammatory microenvironment that is conducive to the repair and regeneration of articular cartilage and subsequent maintenance of the hyaline cartilage phenotype. The down-regulation of Cdkn3 and Cdkn1c genes [33] and the up-regulation of Mki67 gene in RC-D may suggest that the beneficial regulation may be attributed to the fact that chondrocytes in NC-AD being senescent [48] and less proliferative than those in RC-D.

In conclusion, this study developed injectable materials based on collagen type II to repair and regenerate ACDs. After 100 d of repair and regeneration, the histological staining revealed excellent integration of the regenerated tissue with the surrounding native cartilage. The regenerated tissue displayed a hyaline cartilage phenotype, exhibiting biochemical and biomechanical characteristics similar to those of surrounding native cartilage. Gene analysis further demonstrated that compared with the NC-AD, the RC-D repaired with F-Col II+BM+Th showed changes in cartilage-related pathways, as well as down-regulation of TCR signaling pathways and IL-17 signaling pathways, which changed the immune microenvironment of the ACD area. The limitation of this study is that the Negative control, F-Col II+F-gel, and F-Col II+BM groups were not included in the RNA sequencing. If we include all experimental groups, we can compare the repair tissues of each group to clarify the contribution of each component to cartilage repair and regeneration and the related mechanisms.

In conclusion, the successful regeneration of hyaline cartilage using collagen type II-based injectable materials indicated that it could become a new product for clinical conversion and opening a new era of minimally invasive clinical articular cartilage repair.

## Data Availability

The data that support the findings of this study are available in this article and the additional files of this article.
