# Supplementary material for: Collagen Type II-Based Injectable Materials for In situ Repair and Regeneration of Articular Cartilage Defect
Source: Biomater Res. 2024 Aug 30;28:0072. doi: 10.34133/bmr.0072 (PMC11362811; doi:10.34133/bmr.0072)
Supplement: Supplementary 1 — Figs. S1 to S4 Tables S1 to S2 [file bmr.0072.f1.doc]

| Item | Number of legs | Number of rats | Total of rats  (Four group) |
| --- | --- | --- | --- |
| Histology staining after 50 days | 1 | 0.5 | 2 |
| Histology staining and micro-CT after 100 days | 3 | 1.5 | 6 |
| Biochemical assays and biomechanical testing after 100 days | 3 | 1.5 | 6 |
| RNA sequencing after 100 days | 3 | 1.5 | 6 |
| Item | Number of legs | Number of rats | Total of rats  (Two group) |
| Histology staining after 150 days | 1 | 0.5 | 1 |
| Total | 10 | 5 | 21 |

Supplemental Table 1. The number of animals used in this study.

**Supplemental** Table 2. Pineda cartilage repair score

| **Characteristics** | **Score** |
| --- | --- |
| Filling of defect |  |
| 125% | 1 |
| 100% | 0 |
| 75% | 1 |
| 50% | 2 |
| 25% | 3 |
| 0% | 4 |
| Reconstruction of osteochondral junction |  |
| Yes | 0 |
| Almost | 1 |
| Not close | 2 |
| Matrix staining |  |
| Normal | 0 |
| Reduced staining | 1 |
| Significantly reduced staining | 2 |
| Faint staining | 3 |
| No stain | 4 |
| Cell morphology |  |
| Normal | 0 |
| Most hyaline and fibrocartilage | 1 |
| Mostly fibrocartilage | 2 |
| Some fibrocartilages, but mostly nonchondrocytic cells | 3 |


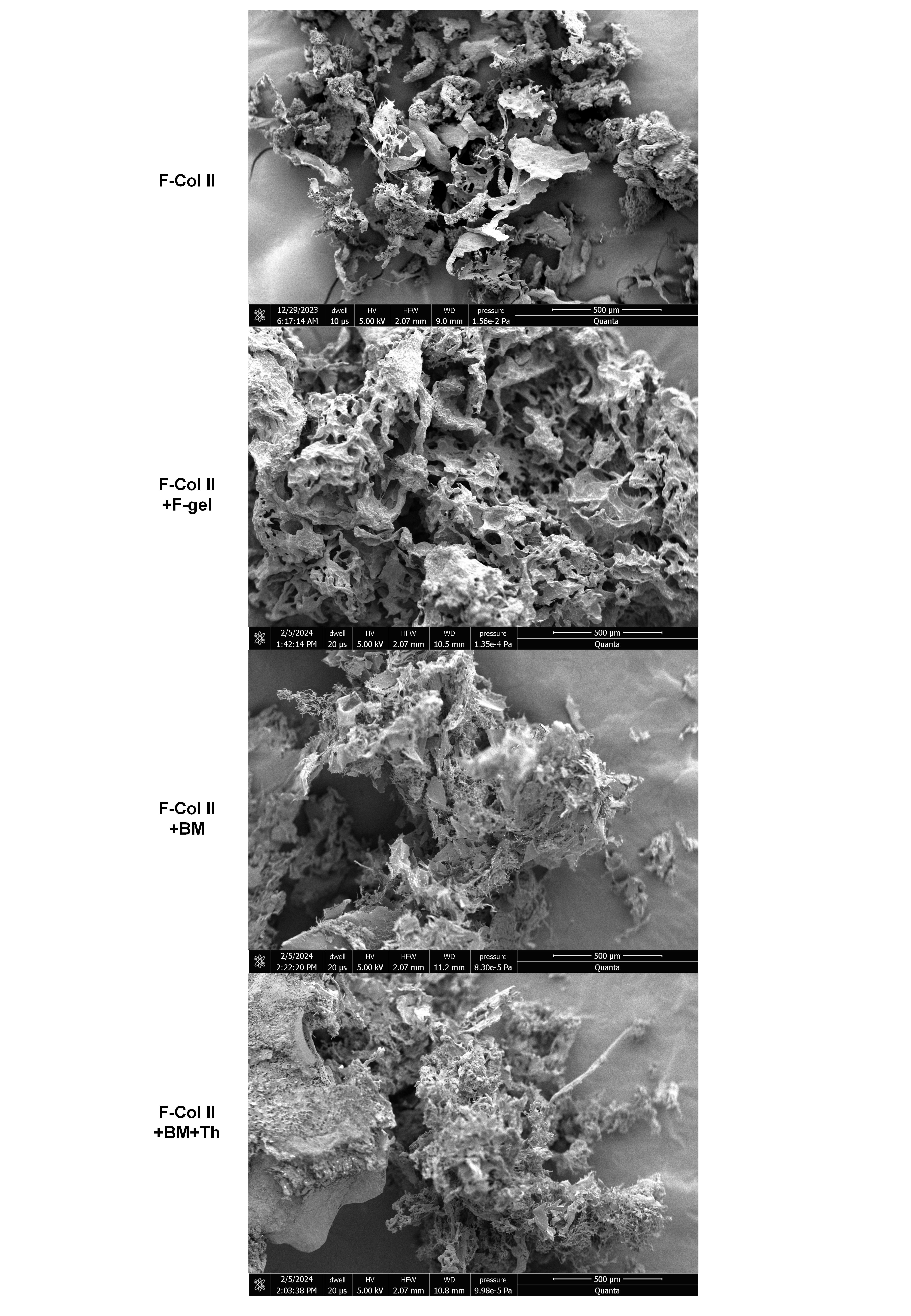


Supplemental Figure 1. SEM of the F-Col II (Image magnified 100 times, the scale bar in the images represents 500 μm).

Supplemental Figure 2. The IHC staining for Col 6 on (A) day 50, (B) day 100, (C) and day 150 after material injection. (D) The staining at day 100 was quantitatively evaluated using Image J software. (n=3.) The scale bar in the images represents 200 μm. Regeneration area (RA), and native area (NA)


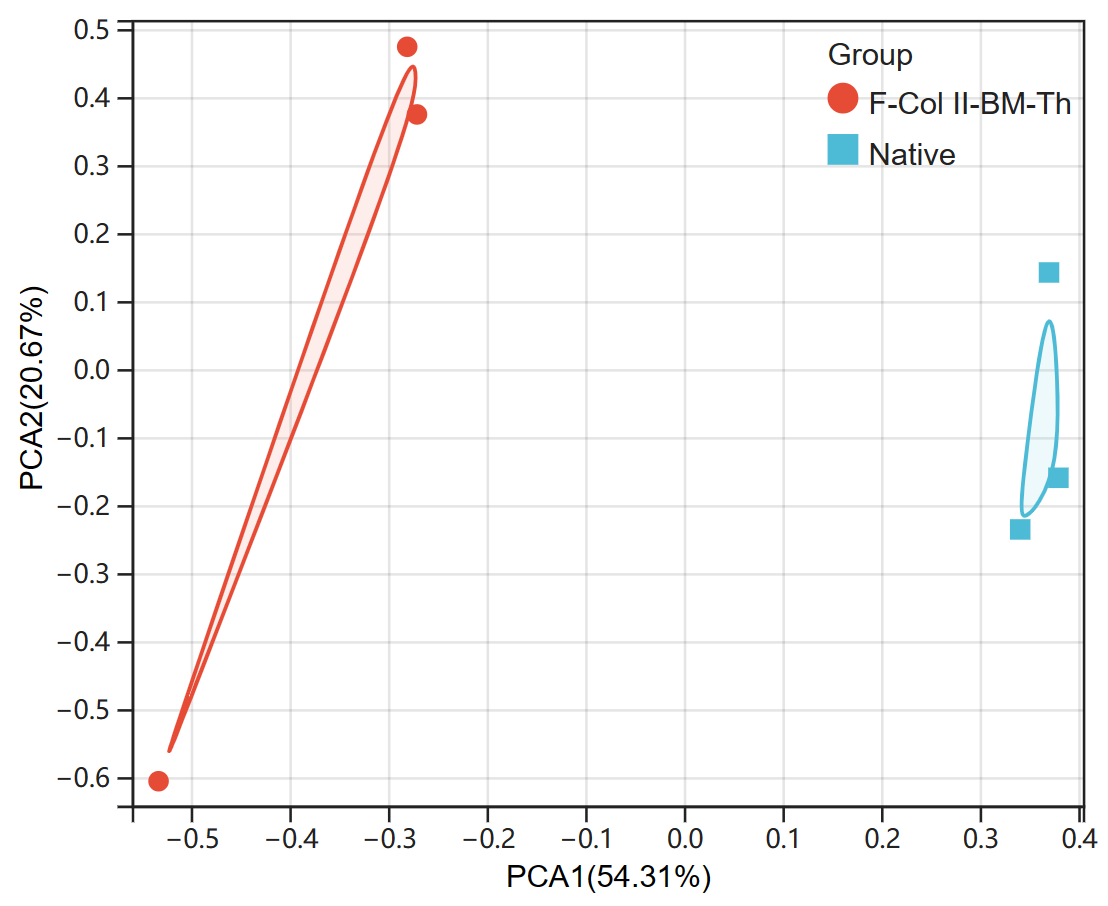


Supplemental Figure 3. Principal component analysis (PCA) revealed a notable separation between the F-Col II-BM-Th and native cartilage tissues.


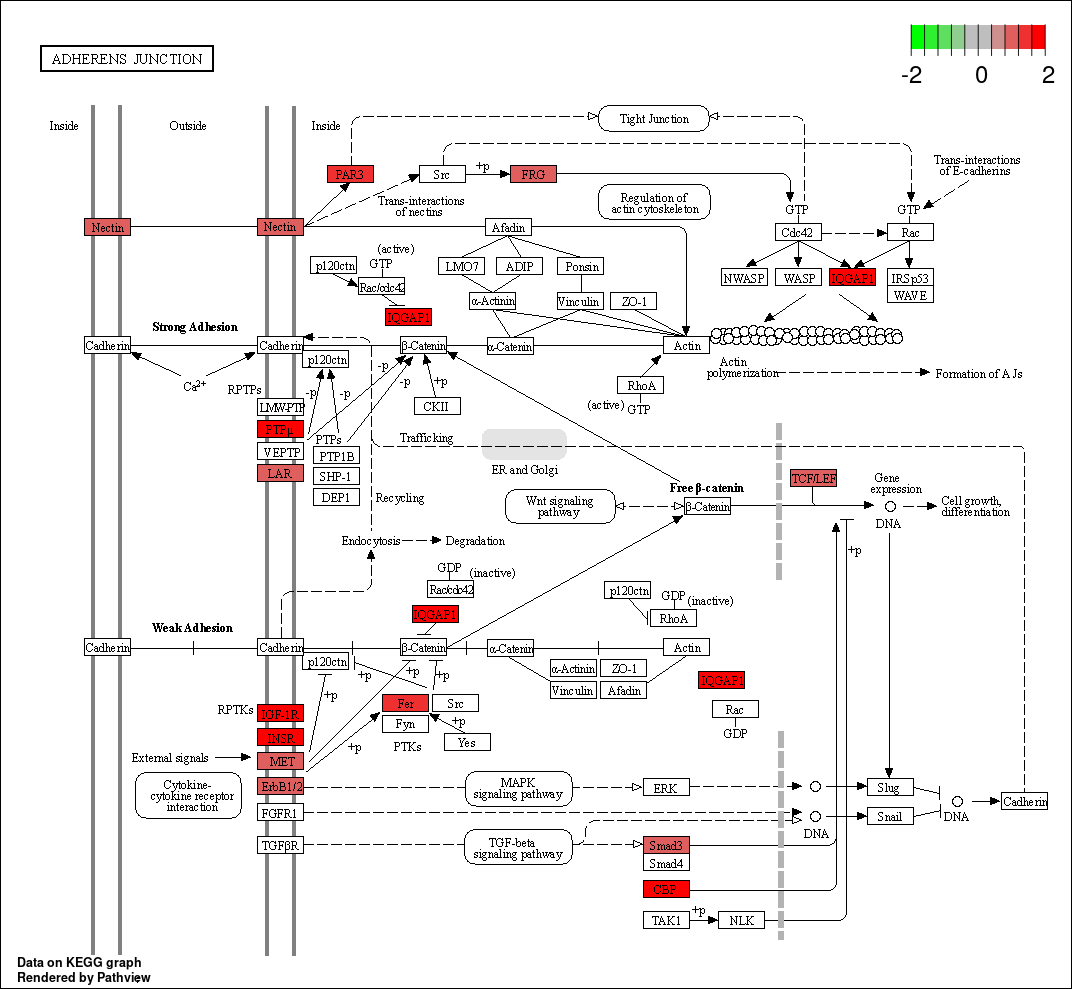


Supplemental Figure 4. The analysis of Adhesion Junction by KEGG.
